# Comparison of Error Rates in Technical Skills and Contextual Skills in a Large-Scale Peer-Assessed Clinical Skills Examination

**DOI:** 10.1007/s40670-026-02764-x

**Published:** 2026-06-03

**Authors:** David Alexander Christian Messerer, Oliver Keis, Astrid Horneffer

**Affiliations:** 1 Institute of Transfusion Medicine, University Hospital Ulm, Ulm, Germany; 2 Office of the Dean of Studies, Medical Faculty, Ulm University, Ulm, Germany

**Keywords:** Medical education, Skills lab, Physical examination, Patient safety

## Abstract

Training of clinical skills often prioritizes technical performance, while contextual skills such as hygienic practice, communication, and documentation receive less systematic attention. In a large-scale peer-assessed examination, third-year medical students completed examination encounters (n = 2,076) using a standardized 20-item binary checklist with predefined allocations to technical and contextual skills. Error rates were low but variable in both categories (technical: 10% (5%; 15%); contextual: 0% (0%; 17%), median with interquartile range). These findings support integrating contextual competence into structured skills education and suggest that brief, checklist-based assessments may reveal actionable deficits in hygiene and documentation that would otherwise remain implicit.

**Supplementary Information:**

The online version contains supplementary material available at https://doi.org/10.1007/s40670-026-02764-x.

## Background

Clinical skills education in undergraduate medicine has traditionally emphasized discrete technical actions, such as focused physical examination maneuvers. Non-technical, contextual elements including hygiene, communication, and documentation have often been taught separately or left implicit [1–3]. Simulation-based formats that are commonly used to teach such contextual competencies are potentially more resource-intensive, requiring dedicated space, equipment, substantial faculty time, and costs – factors that may pose a barrier to broader implementation [4, 5]. Studies of single procedural elements support this view. For example, in peripheral venous cannulation and venipuncture, students frequently perform the core technical motion correctly. In contrast, hygiene sequences such as hand disinfection, preparation of the puncture site, and sterile handling are executed inconsistently or omitted [6].

Even when examinations focus on short, discrete physical examination actions, substantial performance gaps have been documented. Studies of cardiovascular, abdominal, neurologic, and musculoskeletal examinations show that students frequently omit key maneuvers or perform them incorrectly, despite structured teaching and checklists [7, 8]. Despite this, few undergraduate skills-lab studies have directly compared technical and contextual performance within the same structured assessment, and most focus on single procedural tasks rather than short sequences of physical-examination steps [9]. In the present work, hygiene, communication, and documentation were grouped as “contextual skills” because they are behaviors that frame the technical action rather than being part of the motor sequence itself, and because they are frequently addressed separately from technical training in undergraduate curricula.

The present short communication therefore contributes to this literature by quantifying errors in technical skills and contextual skills in a large cohort of medical students, who performed short, focused examination sequences in a standardized, peer-assessed format. Specifically, we addressed two guiding questions: (i) How do error rates in technical skills compare to error rates in contextual skills across six standardized examination sequences? (ii) Within the contextual domain, are errors distributed uniformly or do specific subdomains (hygiene, communication, documentation) show disproportionate vulnerability across stations?

## Activity

This study was conducted within the undergraduate medical program at Ulm University, which follows a traditional subject-based curriculum spanning six years. The investigated training activity took place in the clinical skills laboratory and was embedded in routine practical skills teaching for students in the third year (first clinical year) at “To-Train-U”, Ulm University's clinical skills training center. While it was beyond the scope to assess demographic data, overall attributes of the student body can be assumed to be similar to previous studies [10, 11].

The skills lab team, in alignment with existing learning objectives and in collaboration with the teaching leads of the respective disciplines (e.g., internal medicine, neurology, orthopedics), developed the checklist for each examination sequence to ensure content validity and curricular coherence. The development process comprised an iterative cycle of (i) initial item drafting by the skills-lab team based on established physical-examination guidelines and existing institutional teaching materials, (ii) expert review by the respective discipline leads for content validity, (iii) pilot testing with peer tutors, and (iv) finalization after consensus without changes during the study period. Items were classified a priori as technical or contextual by the skills-lab team in consensus with the discipline leads, with “technical” defined as the motor or cognitive-diagnostic action of the examination sequence itself (e.g., percussion, palpation, reflex testing) and “contextual” defined as the framing behaviors of hygiene, communication with the patient, and documentation of findings. A binary (performed/not performed) scoring format was chosen to support inter-rater agreement under time constraints of a peer-assessed examination; explicit behavioral anchors were provided for each item in the assessor training to standardize the threshold between “performed” and “not performed” (see Supplementary Table 1 for a representative example). This approach reflects a pragmatic assessment design for brief structured performance examinations, while acknowledging the broader literature that checklist-based formats may trade rating simplicity and standardization against reduced sensitivity to qualitative differences in expertise [12].

The checklist was available to students during their skills training but not during the examination. Students were encouraged to practice the examination sequences as often as they wished, supported by structured practice sessions with peer tutors and supervised open-lab times. Each sequence was designed to be completed within 10 min and was assessed using a predefined list of 20 binary checklist items (a representative checklist is provided in Supplementary Table 1) reflecting core technical actions (referred to as technical skills (TS)) and contextual components (henceforth referred to as contextual skills (CS)) including hygienic practice (hygiene, H), communication (C), and documentation (D). The distribution of technical and contextual skills is reported in Table 1. A score of 16/20 points was required to pass the respective examination sequence.

**Table 1:**
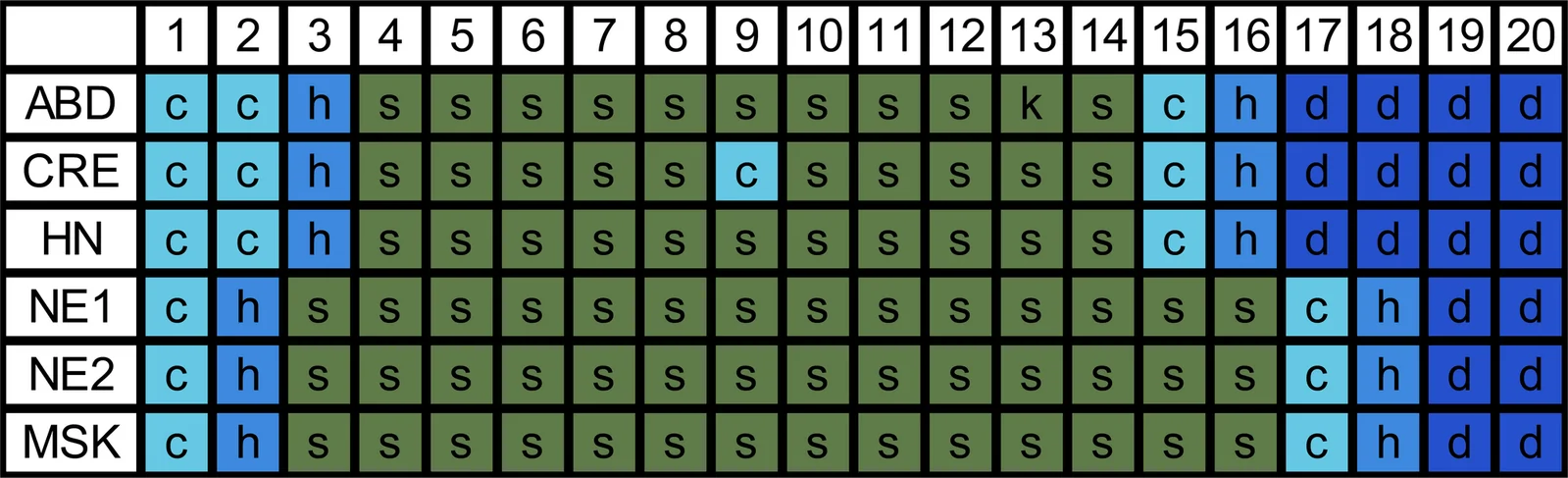
Structure of examinations and distribution of points classified as core technical skills and contextual skills including hygiene (H), communication (C), and documentation (D). Each row lists 20 checklist items per station in order of performance. The letter in each column maps the item to its construct: s = technical skill (“skill”, motor or cognitive-diagnostic action), k = technical skill (“knowledge”, verbal naming of a technical step), h = hygiene, c = communication, d = documentation. Technical items (s, k) range from 7 to 14 per station; contextual items (h, c, d) range from 6 to 10 per station. Examination courses included abdominal examination (ABD), cardiorespiratory examination (CRE), external head-and-neck examination (HN), neurological examination 1 focusing on cranial nerves and reflexes (NE1), neurological examination 2 focusing on motor function, coordination, and sensory testing (NE2), and the musculoskeletal examination of major joints and the spine (MSK)

The skills curriculum is organized in two semesters per academic year. For most students, practical skills training commences in the winter semester and continues in the summer semester. In the winter semester, each student was randomly assigned to complete one of three stations. Students were not informed in advance at which station they would be assessed: abdominal examination (ABD), cardiorespiratory examination (CRE), or external head-and-neck examination (HN). In the summer semester, the examination sequences were: Neurological Examination 1 focused on cranial nerves and reflexes (NE1), Neurological Examination 2 focused on motor function, coordination, and sensory testing (NE2), or the musculoskeletal examination of major joints and the spine (MSK). All examinations were performed on healthy peers of the students.

**Fig. 1:**
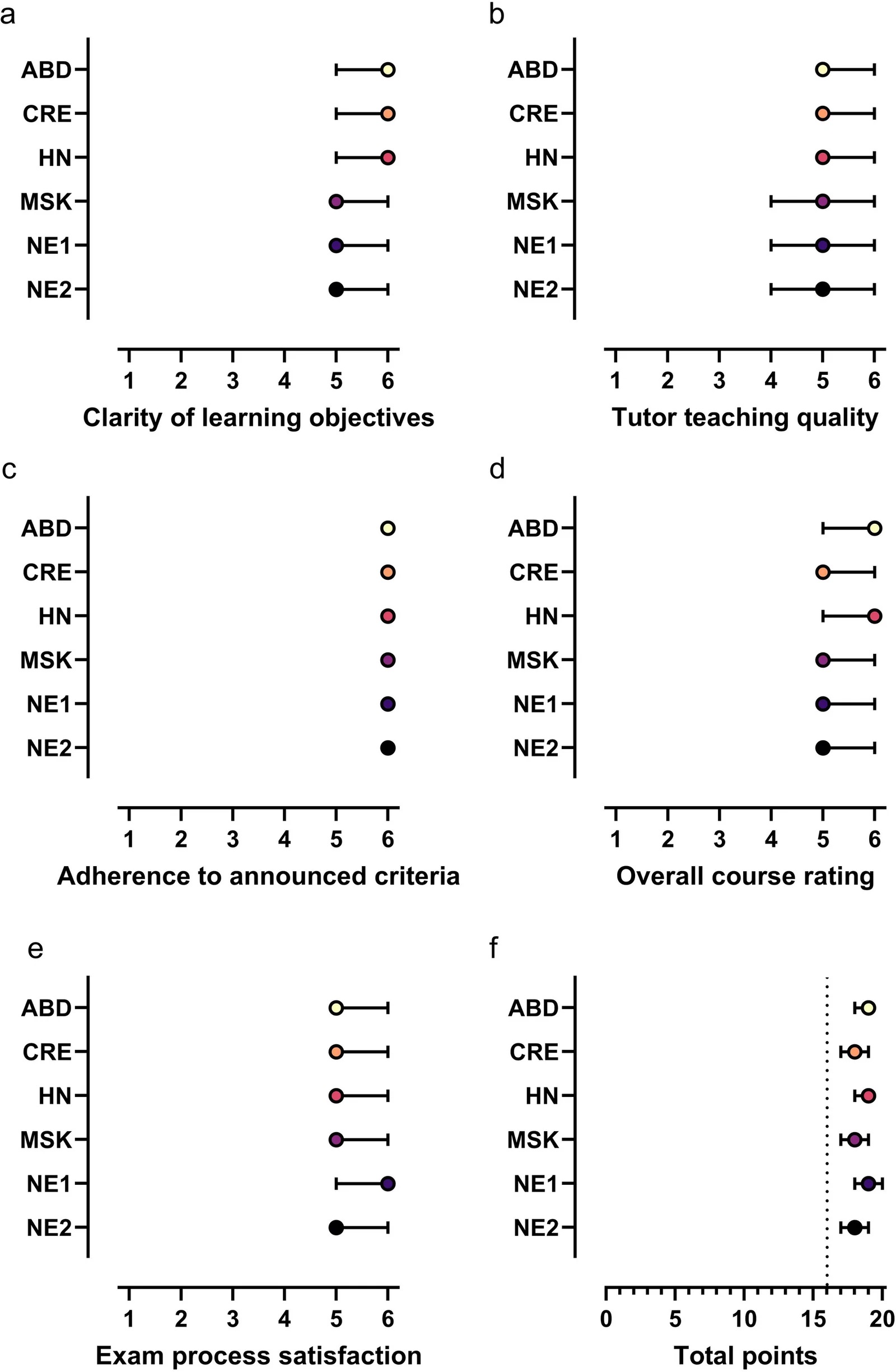
Acceptance and evaluation of the examination stations. Before the examination, students rated **a**) “The learning objectives were clearly formulated” and **b**) “The tutor conveyed the practical skill well” on a 6-point Likert-type scale (1 = strongly disagree, 6 = strongly agree). After the examination but before receiving their results, students rated **c**) “The examination was conducted according to the announced criteria” on the same scale and **d**) “Overall, I would rate the practice station and examination as follows” on a 6-point global rating scale (1 = unsatisfactory, 6 = very good). After the examination, peer assessors rated **e**) “I am satisfied with the conduct of the examination” on a 6-point Likert-type scale (1 = strongly disagree, 6 = strongly agree). **f**) Total points scored by students; the dotted line indicates the passing mark of 16/20 points. Courses included abdominal examination (ABD, n = 339), cardiorespiratory examination (CRE, n = 361), external head-and-neck examination (HN, n = 337), neurological examination 1 focused on cranial nerves and reflexes (NE1, n = 344), neurological examination 2 focused on motor function, coordination, and sensory testing (NE2, n = 348), and musculoskeletal examination of major joints and the spine (MSK, n = 346). Data are reported as median with interquartile range

We included three consecutive cohorts from the academic years 2022, 2023, and 2024. The 2021 cohort was excluded to avoid artifacts from first-time implementation of the assessment format and potential residual effects of the COVID-19 pandemic. The 2,076 assessment encounters analyzed here originated from 1,022 unique students (median of two assessment encounters per unique student). The response rate for feedback on the (Fig. 1a-e) was 99.2% (non-responders were excluded for this data set). Total points (Fig. 1f) and fail rates were available for all encounters. Because each encounter represents a separate semester-specific station assignment, encounters were treated as the primary unit of analysis. For the cross-domain comparison, within-student per-station error percentages were computed separately for the technical and contextual subsets of each station (Table 1). Likert-type data are reported as median and interquartile range because these variables are ordinal and because this summary is less sensitive to outliers and skewed response distributions than the mean.

Students were invited to complete brief anonymized paper-based surveys before and after the assessment; participation was voluntary. The independent ethics committee of Ulm University deemed the study exempt from formal review and issued a waiver on April 16, 2019 for the anonymous aggregate evaluation of students’ performance.

Assessments were conducted by trained peer tutors from advanced study years. The use of trained student assessors is supported by prior work indicating acceptable feasibility and learner acceptance in formative and, in selected settings, even summative OSCE-type assessments, although some studies also note the need for careful calibration and the possibility of systematic differences from faculty ratings [13]. Before acting as assessors, all peer tutors had completed the physical examination course themselves, passed all six practical examinations, and attended a didactic and assessment training program of approximately 8 h, including instruction in checklist use, examiner calibration, and supervised practice ratings. During the assessed session, peer assessors recorded performance in real time on paper-based forms.

## Results and Discussion

Overall, 2,076 assessment encounters were recorded during the observation period. Pass/fail rates as well as student and assessor acceptance were comparable across the examination stations. Students reported high learning objective clarity (median 5 (5;6) on a 6-point Likert-type scale, 1 = strongly disagree, 6 = strongly agree) and high perceived teaching quality of the peer teachers (5 (5;6)). After the examination but prior to receiving their results, students rated adherence to the examination criteria as very high (6 (6;6)). Global evaluations of the practice and examination stations also indicated high satisfaction (5 (5;6), 1 = unsatisfactory, 6 = very good). Peer assessors likewise reported high satisfaction with the examination process (5 (5;6) on a 6-point Likert-type scale). Across all stations, students achieved 18 (17;19) out of 20 points, resulting in an overall pass rate of 96.8% (Fig. 1).

To examine whether performance differed between TS and CS, we compared success and failure rates for both domains. Across stations, examination sequences comprised 7–14 TS points and 6–10 CS points (Table 1). When data were pooled across all stations, students demonstrated a lower error rate in CS (0% (0%; 17%)) than for TS (10% (5%; 15%); *p* < 0.001, Wilcoxon matched-pairs signed-rank test). This aggregate result indicates that contextual competencies are not inherently “unmeasurable” or peripheral; they can be operationalized in short, checklist-based sequences and contribute meaningfully to performance profiles.

**Fig. 2:**
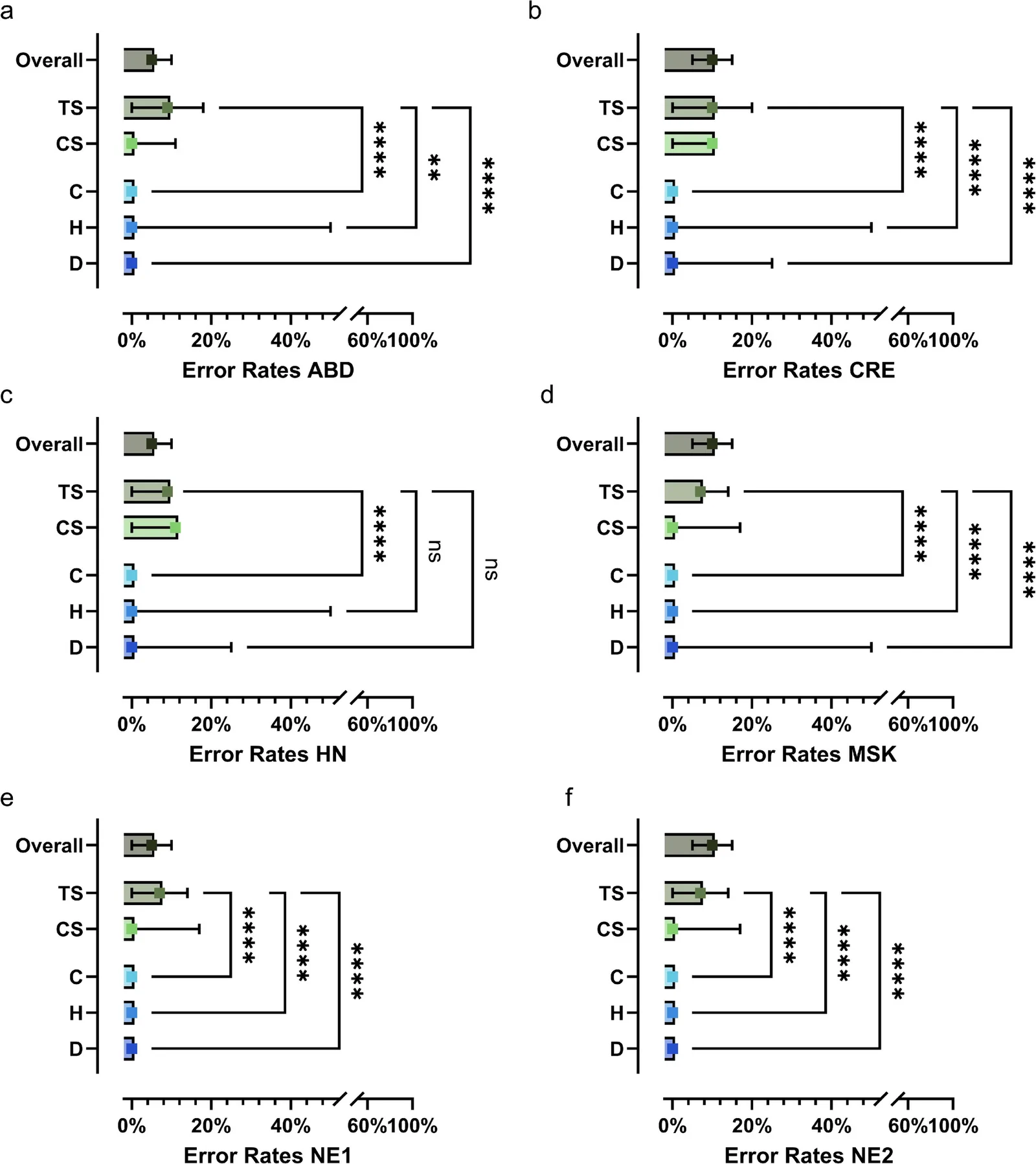
Differences between technical skills (TS) and contextual skills (CS) including hygiene (H), communication (C), and documentation (D). For each panel, the per-encounter percentage error rate is shown for the technical subset (TS) and for each contextual subdomain (H, C, D), calculated as the number of failed items divided by the number of items in that subset within the respective station (denominators as given in Table 1). Reported are results from the courses **a**) abdominal examination (ABD, *n* = 339), **b**) cardiorespiratory examination (CRE, *n* = 361), **c**) external head-and-neck examination (HN, *n* = 337), **d**) musculoskeletal examination of major joints and the spine (MSK, *n* = 346), e) neurological examination 1 focused on cranial nerves and reflexes (NE1, *n* = 344), and f) neurological examination 2 focused on motor function, coordination, and sensory testing (NE2, *n* = 348). Data are reported as median with interquartile range. The x-axis is discontinuous after 60%. Missing error bars indicate identical median and corresponding quartile. ns = not significant, ** and **** denote *p* < 0.01 and *p* < 0.0001, respectively, for prespecified comparisons of TS with C, H, and D using the Friedman test followed by Dunn's multiple-comparisons post hoc correction

However, the station-level pattern shows that this overall advantage of CS does not imply that contextual behavior is uniformly robust. In the detailed per-encounter subdomain analyses, hygiene-related errors showed station-specific vulnerability: the upper quartile of hygiene error rates exceeded 25% in ABD, CRE, and HN. Documentation errors also clustered in selected stations, with the upper quartile of documentation error rates exceeding 20% in CRE and HN (Fig. 2). Thus, the contextual domain was heterogeneous rather than uniform. Although the aggregate CS error rate was low, specific contextual micro-behaviors, particularly hygiene routines and documentation completeness, remained vulnerable in the upper part of the performance distribution under time pressure and task load. This is educationally important, because curricula often treat these domains separately: Technical skills in focused modules, while communication and hygiene are addressed in distinct courses or isolated trainings [1, 2]. The present findings argue against a strict separation: contextual behavior is embedded in routine clinical tasks and can be assessed alongside technical performance, revealing deficits that might otherwise remain invisible until clinical exposure.

For teaching practice, the stability of hygiene and documentation deficits across different stations suggests that at least part of the problem is structural rather than purely task-specific. This supports integrating contextual items early and repeatedly into hands-on training using explicit checklists, immediate feedback, and frequent low-stakes practice. For assessment design, the results support the value of brief, focused examinations: even 10-min sequences can reveal clinically relevant weaknesses in contextual behavior, which may have direct implications for longer simulations and workplace-based assessment strategies.

For educators wishing to integrate contextual items into existing skills lab teaching, three specific aspects are supported by our data. First, contextual items can be embedded in the same checklist as technical items rather than in a separate course, so that hygiene, communication, and documentation are rehearsed in the flow of the examination. Second, the hygiene and documentation subdomains may benefit from explicit, repeatable micro-routines (e.g., fixed pre- and post-examination hand-hygiene moments; a minimal documentation template listing the findings to be recorded) that can be anchored to the clinical task. Third, brief, low-stakes formative assessments with immediate peer feedback may be sufficient to surface the relevant deficits and, in combination with deliberate practice, may offer a pragmatic path toward stabilizing these behaviors.

The present study was not designed to measure downstream patient-safety or economic outcomes, and we therefore restrict our inferences accordingly. Nevertheless, the contextual deficits observed are of a kind that the wider literature has linked to clinically relevant processes. Simulation-based training is resource-intensive [2, 4, 14], and evidence from deliberate practice and mastery learning indicates that stable performance generally requires repeated exposure rather than single assessments [15, 16]. Hand hygiene failures are a major contributor to healthcare-associated infections [17], and inadequate documentation is associated with medication errors, delayed or inappropriate treatment, and compromised continuity of care [18, 19]. Whether repeated contextual-skill-sensitized training during undergraduate education translates into fewer lapses in later clinical practice (“number-needed-to-train”) cannot be answered from our cross-sectional educational dataset and should be tested in longitudinal designs that explicitly link training exposure to clinical process measures.

Several limitations merit consideration. First, although peer assessors were trained and calibrated, peer-based ratings may differ subtly from faculty assessments, particularly for nuanced behaviors that are difficult to score in a binary format. Second, performance was measured in structured skills-lab conditions; error patterns and their consequences may differ in authentic clinical environments. Third, the binary checklist format increases standardization but cannot capture qualitative nuances (e.g., partial completeness of documentation, quality of communication). It supported standardization in a large-scale peer-assessed setting, but likely captured performance breadth more readily than qualitative depth; this is consistent with the broader assessment literature comparing checklist and global rating approaches [12]. Fourth, formal interrater reliability statistics could not be computed because each encounter was rated by a single peer assessor under routine examination conditions. However, the training-based calibration and the fact that the same assessor rated TS and CS within the same encounter mitigate the risk that interrater differences biased the results of the present work. Moreover, the “contextual skills” category is conceptually heterogeneous, so the aggregate CS figure should be interpreted with this heterogeneity in mind rather than as a unified construct. Finally, only focused examination sequences were assessed; findings may not generalize to more complex procedural skills or to later stages of training.

Overall, these findings suggest that contextual competencies can be incorporated into short, structured skills-lab assessments and may reveal concrete, actionable deficits in undergraduate medical education. Within this assessment format, hygiene and documentation emerged as recurring weak points; however, the present study was not designed to establish broader equivalence between contextual and technical skills.

## Supplementary Information

Below is the link to the electronic supplementary material.

Supplementary file1 (DOCX 19 KB)

## Data Availability

All data supporting the findings of this study are available within the article and its Supplementary Information. Original study data may be made available by the corresponding author upon reasonable request, subject to approval by the responsible ethics committee and applicable data protection requirements.
