# Supplementary material for: Comparison of Error Rates in Technical Skills and Contextual Skills in a Large-Scale Peer-Assessed Clinical Skills Examination
Source: Med Sci Educ. 2026 Jun 3;36(4):1703–9. doi: 10.1007/s40670-026-02764-x (PMC13582732; doi:10.1007/s40670-026-02764-x)
Supplement: Supplementary file 1 [file 40670_2026_2764_MOESM1_ESM.docx]

Supplementary Table 1. Representative criterion list for the abdominal examination course (ABD), consisting of 20 binary items, with 80% (16/20 points) required to pass. The instructions (I) were given by the examiner: I-1: “You are to examine the abdomen, liver, spleen, and kidneys of the patient and document your findings. The patient has an unremarkable history and no pain in the area to be examined. Taking a more detailed history is not required in this course.” I-2: “Which two additional examinations belong to a complete abdominal examination?” The criterion list was made available to students before, but not during, the examination and included the following hint: Use the time during hygienic hand disinfection to perform the initial inspection and to explain the upcoming examination steps to the patient.

| **No.** | **I** | **ABD criterion list – 20 points, 10 minutes** |
| --- | --- | --- |
| 1 | I-1 | Greeting the patient and introducing oneself by name and role, clear identification of the patient by actively asking the patient to state their name and date of birth. |
| 2 |  | Ensuring a protected examination environment, announcing the examination,  and asking the patient to expose the abdomen and lie supine and relaxed. |
| 3 | – | Performing hygienic hand disinfection  (nonverbal item: as defined in the course station hand hygiene). |
| 4 | – | Inspecting the abdomen and auscultating bowel sounds separately in all four quadrants. |
| 5 | – | Before percussion/palpation, checking whether the patient has pain or discomfort,  starting the examination in the area where the patient reports the least pain. |
| 6 | – | Percussion performed separately in all four quadrants. |
| 7 | – | Palpation first superficial, then deep in all four quadrants,  while observing the patient’s facial expressions and reactions. |
| 8 | – | Testing for rebound tenderness by deep palpation followed by sudden release while observing for pain on release (while distracting the patient). |
| 9 | – | Correct examination sequence: auscultation before percussion before palpation. |
| 10 | – | Examination of the liver/gallbladder region: palpation below the right costal margin during inspiration and determination of liver borders. |
| 11 | – | Examination of the spleen: approximately 70° right lateral decubitus position, palpation with the right hand under the left costal margin, left hand used as counter-support. |
| 12 | – | Examination of the kidneys: assessment of costovertebral-angle tenderness bilaterally. |
| 13 | I-2 | Naming the two additional examinations:  examination for hernias and digital rectal examination (DRE). |
| 14 | – | Explaining the procedure: hernia examination with the patient standing,  DRE with the patient in the left lateral position, with hips and knees flexed. |
| 15 | – | Verbally ending the examination, asking the patient to get dressed again, saying goodbye and briefly explaining the next steps. |
| 16 | – | Disinfecting the stethoscope (and measuring tape, if used) and performing hygienic hand disinfection (nonverbal item: as defined in the course station hand hygiene). |
| 17 | – | Documentation of patient’s identity and inspection findings. |
| 18 | – | Documentation of auscultation and percussion findings. |
| 19 | – | Documentation of palpation findings. |
| 20 | – | Documentation of liver, spleen, and kidney findings. |
